# Factors related to renal cortical atrophy development after glucocorticoid therapy in IgG4-related kidney disease: a retrospective multicenter study

**DOI:** 10.1186/s13075-016-1175-y

**Published:** 2016-11-25

**Authors:** Ichiro Mizushima, Motohisa Yamamoto, Dai Inoue, Shinichi Nishi, Yoshinori Taniguchi, Yoshifumi Ubara, Shoko Matsui, Tetsuhiko Yasuno, Hitoshi Nakashima, Hiroki Takahashi, Kazunori Yamada, Hideki Nomura, Masakazu Yamagishi, Takao Saito, Mitsuhiro Kawano

**Affiliations:** 1 Division of Rheumatology, Department of Cardiovascular and Internal Medicine, Kanazawa University Graduate School of Medicine, Takara-machi 13-1, Kanazawa, Ishikawa 920-8640 Japan; 2 Division of Nephrology and Rheumatology, Department of Internal Medicine, Ishikawa Prefectural Central Hospital, Kanazawa, Japan; 3 The First Department of Internal Medicine, Sapporo Medical University, South 1, West 16, Chuo-ku, Sapporo, Hokkaido 060-8543 Japan; 4 Department of Radiology, Kanazawa University Graduate School of Medical Science, 13-1 Takara-machi, Kanazawa, Ishikawa 920-8640 Japan; 5 Division of Nephrology and Kidney Center, Kobe University Graduate School of Medicine, 7-5-1 Kusunoki, Chuo-ku, Kobe, Hyogo 650-0017 Japan; 6 Department of Endocrinology, Metabolism and Nephrology, Kochi Medical School, Kochi University, Kohasu, Oko-cho, Nankoku, Kochi 783-8505 Japan; 7 Nephrology Center and Okinaka Memorial Institute for Medical Research, Toranomon Hospital, Toranomon 2-2-2, Minato-ku, Tokyo, 105-8470 Japan; 8 Health Administration Center, University of Toyama, 2630 Sugitani, Toyama-shi, Toyama 930-0194 Japan; 9 Division of Nephrology and Rheumatology, Department of Internal Medicine, Fukuoka University, 7-45-1, Nanakuma, Jonan-ku, Fukuoka 814-0180 Japan; 10 Department of General Medicine, Kanazawa University Hospital, 13-1 Takara-machi, Kanazawa, Ishikawa 920-8640 Japan; 11 Division of Cardiology, Department of Cardiovascular and Internal Medicine, Kanazawa University Graduate School of Medicine, 13-1 Takara-machi, Kanazawa, Ishikawa 920-8640 Japan

**Keywords:** IgG4-related disease, IgG4-related kidney disease, Atrophy, Glucocorticoid

## Abstract

**Background:**

In immunoglobulin G4-related kidney disease (IgG4-RKD), focal or diffuse renal cortical atrophy is often observed in the clinical course after glucocorticoid therapy. This study aimed to clarify the factors related to renal atrophy after glucocorticoid therapy in IgG4-RKD.

**Methods:**

We retrospectively evaluated clinical features including laboratory data and computed tomography (CT) findings before and after glucocorticoid therapy in 23 patients diagnosed with IgG4-RKD, all of whom were followed up for more than 24 months.

**Results:**

Seventeen patients were men, and six were women (average age 62.0 years). Average follow-up period was 54.9 months. The average estimated glomerular filtration rate (eGFR) at diagnosis was 81.7 mL/min/1.73 m^2^. All patients had had multiple low-density lesions on contrast-enhanced CT before glucocorticoid therapy, and showed disappearance or reduction of these lesions after it. Pre-treatment eGFR and serum IgE level in 11 patients in whom renal cortical atrophy developed 24 months after the start of glucocorticoid therapy were significantly different from those in 12 patients in whom no obvious atrophy was found at that time (68.9 ± 30.1 vs 93.5 ± 14.1 mL/min/1.73 m^2^, *P* = 0.036, and 587 ± 254 vs 284 ± 263 IU/mL, *P* = 0.008, respectively). Pre-treatment eGFR and serum IgE level were also significant risk factors for renal atrophy development 24 months after the start of therapy with an odds ratio of 0.520 (per 10 mL/min/1.73 m^2^, 95% confidence interval (CI) 0.273–0.993, *P* = 0.048) and 1.090 (per 10 IU/mL, 95% CI: 1.013–1.174, *P* = 0.022), respectively, in age-adjusted, sex-adjusted, serum IgG4 level-adjusted logistic regression analysis. Receiver operating characteristic curve analysis showed that eGFR of less than 71.0 mL/min/1.73 m^2^ and serum IgE of more than 436.5 IU/mL were the most appropriate cutoffs and yielded sensitivity of 63.6% and specificity of 100%, and sensitivity of 90.9% and specificity of 75.0%, respectively, in predicting renal atrophy development.

**Conclusions:**

This study suggests that pre-treatment renal insufficiency and serum IgE elevation predict renal atrophy development after glucocorticoid therapy in IgG4-RKD.

**Electronic supplementary material:**

The online version of this article (doi:10.1186/s13075-016-1175-y) contains supplementary material, which is available to authorized users.

## Background

Immunoglobulin G4 (IgG4)-related disease (IgG4-RD) is a recently recognized systemic fibro-inflammatory disorder that can affect almost all organs in the body [1, 2]. It frequently causes various renal lesions, which are collectively referred to as IgG4-related kidney disease (IgG4-RKD) [3]. Many studies have clarified the clinical, radiographic, and histopathological features of this disease [3–6].

Clinical and histopathological responses to glucocorticoid therapy in IgG4-RKD have been characterized by several recent studies [7–10], and the prognosis of this disease is not always favorable. Although glucocorticoid therapy results in a rapid improvement of renal function and radiologic findings within one month after the start of therapy in most cases of IgG4-RKD, recovery of renal function is suboptimal in the patients with moderate to severe renal dysfunction before therapy [8]. Moreover, in the clinical course after glucocorticoid therapy, focal or diffuse renal cortical atrophy is observed in a considerable proportion of treated patients. However, the factors related to such renal atrophy have not been well-clarified. This state of affairs prompted us to undertake the present study to clarify the factors related to renal atrophy after glucocorticoid therapy in IgG4-RKD.

## Methods

### Patients and materials

Between 1 January 2007 and July 2015, 27 patients with IgG4-RD were enrolled as candidates for this study from seven collaborating institutions in Japan. Among them, we identified 23 patients with IgG4-RKD with typical renal radiological findings, whose follow-up period was more than 24 months, and who had sufficient follow-up data (Table 1). We diagnosed them as having IgG4-RKD based on their fulfillment of the criteria proposed by the Japanese Society of Nephrology [3] and exclusion of other diseases. All patients had IgG4-related involvement of more than one extra-renal organ.

**Table 1:** Baseline clinical characteristics of 23 patients with IgG4-related kidney disease

Characteristic	Value (n = 23 patients)
Age, years	62.0 ± 12.0
Gender, male (%)	73.9
Follow-up period (months)	54.9 ± 22.8
Allergy (%)	65.2
Number of extra-renal organs	3.0 ± 1.3
IgG4 (mg/dL)	1069 ± 533
IgG (mg/dL)	3021 ± 1243
IgE (IU/mL)	429 ± 296
Hypocomplementemia (%)	34.8
CRP (mg/dL)	0.25 ± 0.35
Cr (mg/dL)	1.00 ± 0.48
eGFR (mL/min/1.73 m^2^)	81.7 ± 25.8
Initial dose of PSL (mg/kg/day)	0.57 ± 0.17
Other immunosuppressants (%)	21.7
ARB (%)	21.7
ACEI (%)	0
Relapse (%)	34.8
Diabetes mellitus (%)	30.4
Hypertension (%)	21.7
Ischemic heart disease (%)	4.3
Cerebral vascular disease (%)	0
Smoking habit (%)	52.4

Conversion factor for serum creatinine at diagnosis (*Cr*) mg/dL to μmol/L, ×88.4. *ACEI* angiotensin converting enzyme inhibitor, *ARB* angiotensin II receptor blocker, *CRP* serum C-reactive protein at diagnosis, *eGFR* estimated glomerular filtration rate at diagnosis, *IgG* serum immunoglobulin G at diagnosis, *IgG4* serum immunoglobulin G4 at diagnosis, *IgE* serum immunoglobulin E at diagnosis, *PSL* prednisolone

Renal biopsy was performed in 11 patients, and biopsy of the affected extra-renal organs including salivary and lacrimal glands in the other 10 patients. All of these 21 patients were diagnosed with definite IgG4-RKD with typical histopathological and immunohistochemical findings. The remaining two patients fulfilled the diagnostic criteria of autoimmune pancreatitis (AIP) [11] and IgG4-related dacryoadenitis and sialoadenitis (IgG4-DS) [12], respectively, although they were diagnosed with possible IgG4-RKD. We retrospectively evaluated the clinical features including laboratory data and imaging findings at baseline and during the follow-up period in these 23 patients. The clinical course of all patients was compatible with that of typical IgG4-RD patients, including an initial good response to glucocorticoid. Five patients (patients 3, 9, 15, 19, and 20 in Additional file 1: Table S1) had been included in our earlier study [13] and another four patients (patients 13, 17, 22, and 23 in Additional file 1: Table S1) in another earlier study [7].

### Imaging evaluation

All patients underwent whole-body computed tomography (CT) examination at the initial diagnosis, and had evidence of multiple low-density lesions on contrast-enhanced CT. Follow-up CT data were available for all 23 patients, who received glucocorticoid therapy. A single radiologist with extensive experience in IgG4-RD at Kanazawa University Hospital, who was blinded to other clinical data including renal function, reviewed all imaging data.

The sites of renal and extra-renal involvement were noted at the initial diagnostic CT imaging, and atrophic change development, improvement of individual pre-existing renal lesions, and appearance of any new renal lesions were examined at follow-up CT imaging including images within 24 months after the start of treatment and at the last review. All subsequent CT scans within 24 months were routinely performed to follow the clinical course and ascertain the efficacy of treatment, while those from 24 months after the start of treatment to the last review were undertaken for various reasons including routine monitoring or specific clinical indications such as suspected relapse.

Atrophic change in renal lesions was defined as a local dimple in the renal cortex or decrease in the total renal volume compared with the initial baseline scan, as judged by the radiologist, who took careful consideration of the age and constitution of each patient. Radiologic improvement of renal lesions was defined as recovery of renal cortical contrast enhancement without atrophic change as mentioned above at the same site as the low-density lesion detected at the initial diagnosis.

### Definition of clinical improvement and relapse

Clinical improvement in IgG4-RKD was defined as stabilization or improvement in renal function in terms of serum creatinine level or estimated glomerular filtration rate (eGFR) and radiologic improvement as mentioned above. Improvement in extra-renal lesions was identified according to changes in symptomatic, radiologic, serologic, and/or histologic features [14, 15]. Relapse of IgG4-RKD was identified by each attendant physician on the basis of a rapid rise in serum creatinine after careful exclusion of other renal diseases, and/or reappearance or worsening of the radiologic findings. Relapse of extra-renal lesions was defined as reappearance or worsening of symptomatic, radiologic, serologic, or histologic features [14, 15]. In IgG4-RD, re-elevation of serological values such as serum IgG or IgG4 without clinical symptoms or abnormal radiologic findings was not considered as relapse.

### Statistical analysis

Statistical analysis was performed using SPSS V.19. Data are presented as means ± standard deviation. The significance of differences between groups was determined using the Mann-Whitney *U* test or Wilcoxon signed rank test, while the significance of differences in frequencies was analyzed with Fisher’s exact probability test. For assessment of risk factors for development of atrophy 24 months after the start of therapy, unadjusted and age-adjusted, sex-adjusted, serum IgG4 level-adjusted logistic regression analyses were conducted. Receiver operating characteristic (ROC) curve analysis was performed to test the usefulness of certain parameters for the prediction of renal cortical atrophy and to determine the appropriate cutoff value. Significant differences were defined by *P* < 0.05.

## Results

### Baseline patient profiles

Baseline clinical characteristics of the 23 patients with IgG4-RKD and typical renal radiological findings are listed in Table 1. There were 17 men and 6 women with an average age of 62 ± 12 years (range 34–77). One patient (patient 8 in Additional file 1: Table S1) had been treated with prednisolone at a dose of 7 mg/day for IgG4-DS. None of the other 22 patients had been treated with any immunosuppressant drugs, including glucocorticoids, before the diagnosis of IgG4-RKD. All patients had involvement of one or more extra-renal organs (average 3.0 ± 1.3 organs, range 1–7). The salivary gland was involved in 20 patients (87.0%), the lacrimal gland in 14 patients (60.9%), the pancreas, a perivascular lesion, and the lung in 8 patients respectively for each (34.8%), the retroperitoneum in 3 patients (13.0%), the prostate and hepatic-biliary tract in 2 patients respectively for each (8.7%), and a nerve in 1 patient (4.3%). The mean follow-up period of the 23 patients after diagnosis was 54.9 ± 22.8 months (range 28–93).

At presentation, all patients had elevated serum IgG4 (average 1069 ± 533 mg/dL, range 263–2160, normal range <105), which was detected using the assay methods of nephelometry, and IgG (average 3021 ± 1243 mg/dL, range 1756–6729, normal range 870–1700). There were 16 patients (69.6%) with elevated serum IgE (average 429 ± 296 IU/mL, range 8–1226, normal range <250): 8 patients (34.8%) had hypocomplementemia. The average serum creatinine level was 1.00 ± 0.48 mg/dL (range 0.40–2.55), and 10 patients (43.5%) had elevated serum creatinine exceeding 1.0 mg/dL. The average eGFR calculated on the basis of the Chronic Kidney Disease Epidemiology Collaboration (CKD-EPI) equations, which provide the best estimate for individuals with normal or mildly reduced eGFR [16], was 81.7 ± 25.8 mL/min/1.73 m^2^ (range 17.8 –116.6), and 3 patients (13.0%) had eGFR <60 mL/min/1.73 m^2^. Average serum C-reactive protein (CRP) was 0.25 ± 0.35 mg/dL (range 0.0–1.2), and only 2 (8.7%) of 23 patients had elevated serum CRP (CRP >1 mg/dL) (Table 1) (see Additional file 1 for more detail on these data).

**Fig. 1:**
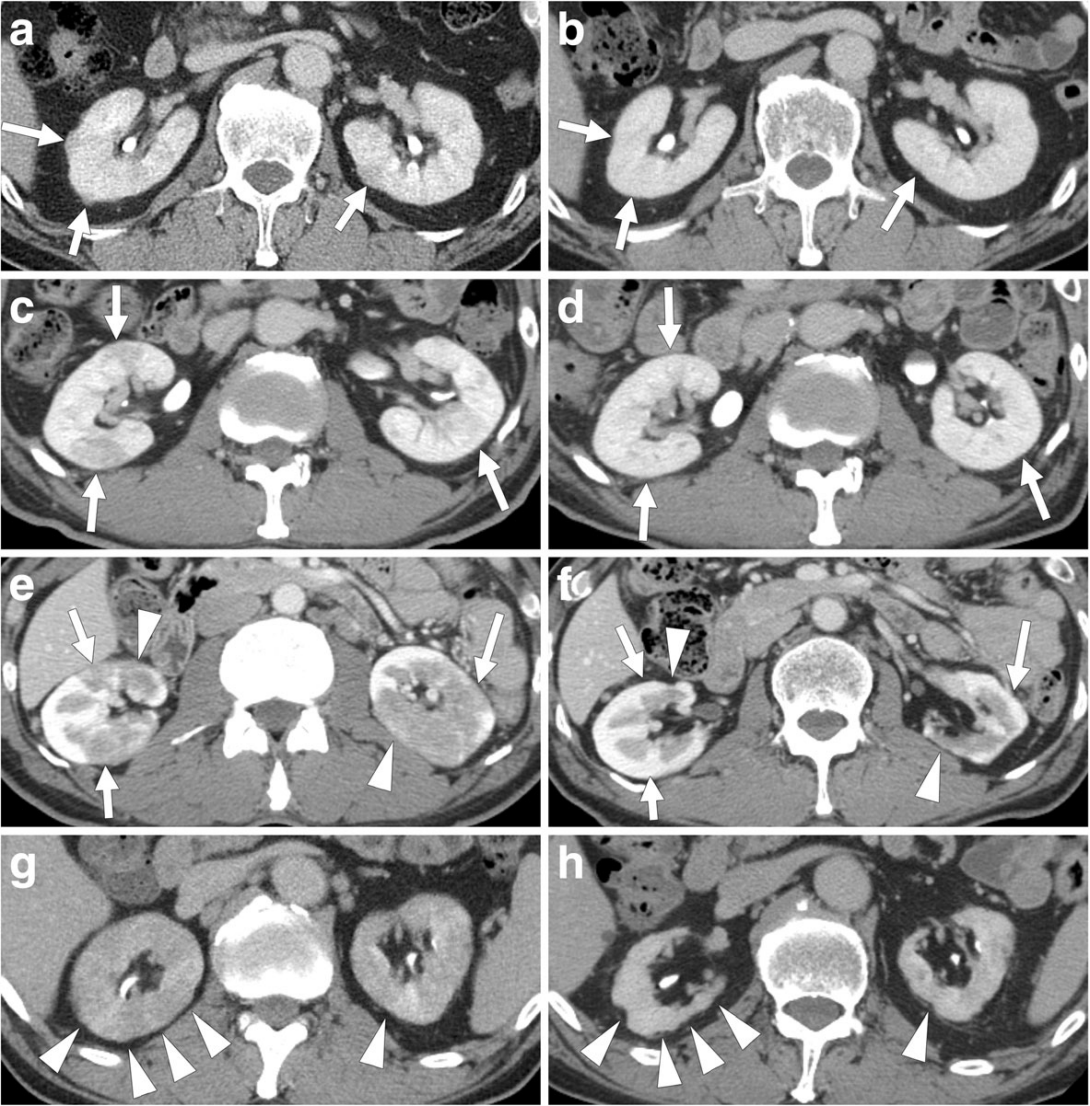
Low-density lesions after glucocorticoid therapy. The outcome of low-density lesions after glucocorticoid therapy varied between individual patients and even between individual lesions: **a**, **c**, **e**, **g** pre-treatment; **b**, **d**, **f**, **h** post-treatment. Representative cases are shown: patient 10 (**a**, **b**); patient 15 (**c**, **d**); patient 17 (**e**, **f**); patient 22 (**g**, **h**). Patient 10 had small peripheral cortical nodules. Patients 15 and 17 had multiple, round or wedge-shaped lesions. Patient 22 had diffuse patchy involvement. *Arrows* show recovering lesions, and *arrowheads* show atrophic lesions

CT revealed renal parenchymal low-density lesions in all patients (Fig. 1). These lesions were patchily distributed hypo-attenuated lesions in the renal parenchyma, and were single or multiple, and round or wedge-shaped. Of the 23 patients, 3 also had diffuse thickening of the renal pelvic wall characterized by a smooth intra-luminal surface. CT also revealed typical extra-renal lesions mainly in the salivary glands, lacrimal glands, pancreas, periaortic/periarterial tissue, and lung.

Diabetes mellitus (DM) was present in seven patients, hypertension (HT) in five, ischemic heart disease in only one; none of the patients had cerebral vascular disease. Information on smoking history was available for 21 patients (52.4%), of whom 11 had a past or current smoking habit.

### Treatment

The indications for treatment and the treatment regimen were decided by the respective attending physician. All 23 patients were treated with prednisolone at an average initial dose of 35.7 ± 8.3 mg/day (range 20–50) ([0.57 ± 0.17 mg/kg/day (range 0.28–0.97)) after the diagnosis. The initial prednisolone dose was continued until 2 to 5 weeks after the start of therapy, and gradually tapered to 2.5–9.0 mg/day in all cases. The average prednisolone dose at the last review was 6.0 ± 1.7 mg/day. Eighteen patients were treated with glucocorticoid alone during the clinical course, and the remaining five patients with a combination of glucocorticoid and other immunosuppressant drugs such as azathioprine, tacrolimus, or cyclosporine. An angiotensin II receptor blocker (ARB) was administered to five patients for the treatment of hypertension during the clinical course; none of the patients received any angiotensin converting enzyme inhibitors (ACEI).

### Clinical and radiological course after glucocorticoid therapy

After glucocorticoid therapy, all patients had CT evidence of disappearance or reduction of the low-density lesions during follow up (Fig. 1). Some of the lesions resulted in renal cortical atrophy 24 months after the start of therapy in 11 patients (group A), whereas no obvious atrophy was found at that time in 12 patients (group B).

**Fig. 2:**
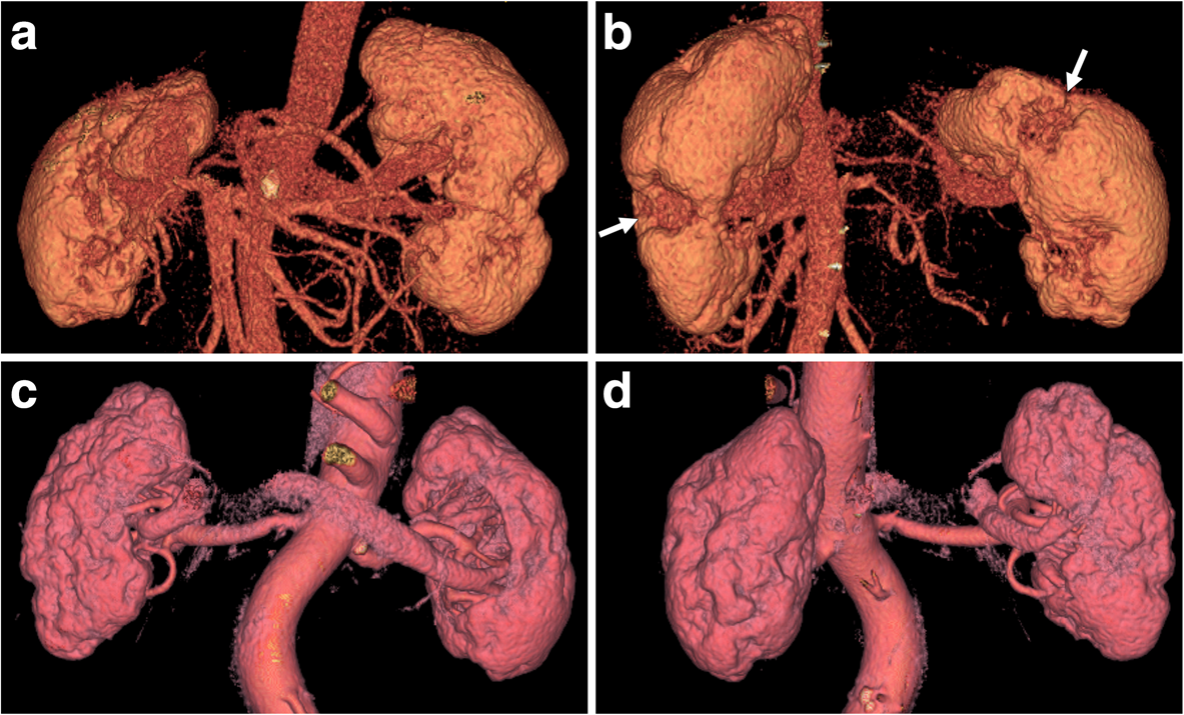
Three-dimensional computed tomography (CT). Three-dimensional CT revealed the appearance of multiple renal cortical scars resembling craters. Patient 22 (**c**, **d**) with worse pre-treatment estimated glomerular filtration rate (35.4 mL/min/1.73 m^2^) than patient 17 (69.3 mL/min/1.73 m^2^) (**a**, **b**) had many more cortical scars. *Arrows* show atrophic lesions

In five patients treated with glucocorticoid and another immunosuppressant drug during the treatment course, four patients experienced relapse, and one patient had renal atrophy 24 months after the start of therapy. In group A, the mean duration between initiation of treatment and detection of atrophy was 8.9 ± 5.4 months. Diffuse thickening of the renal pelvic wall became thinner or normalized after therapy in all of three patients. No development of new renal lesions was observed in any patients under maintenance glucocorticoid therapy during follow up. Three-dimensional (3D) CT was performed in two patients after successful glucocorticoid therapy, and revealed the development of multiple renal cortical scars resembling craters (Fig. 2).

The average eGFR in all 23 patients (81.7 ± 25.8 mL/min/1.73 m^2^ before therapy) changed to 85.9 ± 20.7 at 1 month after the start of therapy, 87.6 ± 18.0 at 12 months, and 82.0 ± 17.3 at the last review. In group A, the average eGFR of 68.9 ± 30.1 mL/min/1.73 m^2^ before therapy changed to 77.5 ± 22.2 at 1 month after the start of therapy, 81.8 ± 22.0 at 12 months, and 78.2 ± 21.1 at the last review. In group B, the average eGFR of 93.5 ± 14.1 mL/min/1.73 m^2^ before therapy changed to 93.4 ± 16.6 at 1 month after the start of therapy, 93.3 ± 12.2 at 12 months, and 85.5 ± 13.0 at the last review.

### Factors related to renal cortical atrophy after glucocorticoid therapy

To identify the factors related to renal atrophy after glucocorticoid therapy, pre-treatment clinical characteristics including co-morbidities, prednisolone dose, other co-medication including other immunosuppressant drugs and ARB, and relapse rate were compared between groups A and B (Table 2). Pre-treatment eGFR in group A was significantly lower than that in group B (68.9 ± 30.1 vs 93.5 ± 14.1 mL/min/1.73 m^2^, *P* = 0.036). Pre-treatment serum IgE in group A was significantly higher than that in group B (587 ± 254 vs 284 ± 263 IU/mL, *P* = 0.008). None of the other factors significantly differed between the two groups.

**Table 2:** Comparison of patients with and without renal atrophy 24 months after the start of glucocorticoid therapy

	Atrophy (+) (n = 11)	Atrophy (-) (n = 12)	*P* value
Age (years)	62.6 ± 10.7	61.6 ± 13.5	0.951
Gender (male, %)	72.7	75.0	1.000
Allergy (%)	54.5	75.0	0.400
Number of extra-renal organs	2.8 ± 1.0	3.3 ± 1.6	0.423
IgG4 (mg/dL)	909 ± 587	1216 ± 454	0.065
IgG (mg/dL)	3001 ± 1505	3040 ± 1015	0.424
IgE (IU/mL)	587 ± 254	284 ± 263	0.008
CH50 (IU/L)	31.0 ± 20.5	34.9 ± 14.8	0.498
CRP (mg/dL)	0.31 ± 0.38	0.19 ± 0.32	0.251
Cr (mg/dL)	1.23 ± 0.59	0.80 ± 0.20	0.052
eGFR (mL/min/1.73 m^2^)	68.9 ± 30.1	93.5 ± 14.1	0.036
Initial dose of PSL (mg/kg/day)	0.52 ± 0.18	0.62 ± 0.16	0.124
Other immunosuppressant drugs (%)	9.1	33.3	0.317
ARB (%)	27.3	16.7	0.640
Relapse (%)	18.2	50.0	0.193
Diabetes mellitus (%)	36.4	25.0	0.667
Hypertension (%)	27.3	16.7	0.640
Ischemic heart disease (%)	0	8.3	1.000
Smoking habit (%)	50.0	54.5	1.000

Conversion factor for serum creatinine at diagnosis (Cr) mg/dL to μmol/L, ×88.4. *ARB* angiotensin II receptor blocker, *CRP* serum C-reactive protein at diagnosis, *eGFR* estimated glomerular filtration rate at diagnosis, *IgG* serum immunoglobulin G at diagnosis, *IgG4* serum immunoglobulin G4 at diagnosis, *IgE* serum immunoglobulin E at diagnosis, *PSL* prednisolone

To evaluate the relationship of pre-treatment eGFR and serum IgE level with development of renal atrophy 24 months after the start of therapy, logistic regression analyses were performed for various demographic and clinical variables at baseline. Pre-treatment eGFR and serum IgE level were significant risk factors for development of renal atrophy 24 months after the start of therapy with an odds ratio (OR) of 0.520 (per 10 mL/min/1.73 m^2^, 95% CI 0.273–0.993, *P* = 0.048) and 1.090 (per 10 IU/mL, 95% CI 1.013–1.174, *P* = 0.022), respectively, in age-adjusted, sex-adjusted, serum IgG4 level-adjusted logistic regression analysis (Table 3).

**Table 3:** Odds ratio for risk of renal atrophy development 24 months after the start of therapy: unadjusted and age-adjusted, sex-adjusted, serum IgG4 level-adjusted logistic regression

Variable	Unadjusted			Age-adjusted, sex-adjusted, serum IgG4 level-adjusted		
	OR	95% CI	*P* value	OR	95% CI	*P* value
Age (per year)	1.007	0.939 to 1.080	0.844	1.023	0.948 to 1.104	0.553
Male sex	0.889	0.138 to 5.723	0.901	2.045	0.228 to 18.311	0.522
Allergy	0.400	0.068 to 2.337	0.309	0.327	0.040 to 2.674	0.297
Serum IgG4 concentration at baseline (per 10 mg/dL)	0.988	0.970 to 1.005	0.175	0.983	0.963 to 1.005	0.125
Serum IgG concentration at baseline (per 10 mg/dL)	1.000	0.993 to 1.006	0.938	1.006	0.995 to 1.017	0.270
Serum IgE concentration at baseline (per 10 IU/mL)	1.056	1.004 to 1.111	0.035	1.090	1.013 to 1.174	0.022
Serum C3 concentration at baseline (per mg/dL)	1.001	0.977 to 1.025	0.957	0.995	0.967 to 1.024	0.718
Serum C4 concentration at baseline (per mg/dL)	0.987	0.896 to 1.088	0.795	0.982	0.881 to 1.094	0.740
Serum CH50 titer at baseline (per IU/L)	0.987	0.940 to 1.036	0.585	0.980	0.928 to 1.034	0.452
Serum CRP concentration at baseline (per mg/dL)	2.776	0.208 to 37.079	0.440	6.149	0.273 to 138.380	0.253
eGFR at baseline (per 10 mL/min/1.73 m^2^)	0.600	0.365 to 0.987	0.043	0.520	0.273 to 0.993	0.048
Number of organs involved	0.715	0.634 to 1.436	0.350	0.793	0.330 to 1.910	0.606
Initial PSL dose (per mg/kg/day)	0.022	0.000 to 6.131	0.184	0.050	0.000 to 54.982	0.402
Other immunosuppressant drugs	0.200	0.019 to 2.162	0.185	0.140	0.010 to 2.041	0.150
ARB	1.875	0.250 to 14.082	0.541	1.008	0.088 to 11.573	0.995
Diabetes mellitus	1.714	0.285 to 10.383	0.556	1.813	0.191 to 17.236	0.605
Hypertension	1.875	0.250 to 14.082	0.541	0.908	0.070 to 11.815	0.941
Smoking habit	0.833	0.150 to 4.636	0.835	0.652	0.091 to 4.655	0.669

Conversion factor for creatinine (Cr): mg/dL to μmol/L, ×88.4. *ARB* angiotensin II receptor blocker, *CI* confidence interval, *CRP* C-reactive protein, *eGFR* estimated glomerular filtration rate, *OR* odds ratio, *IgG* immunoglobulin G, *IgG4* immunoglobulin G4, *IgE* immunoglobulin E, *PSL* prednisolone

**Fig. 3:**
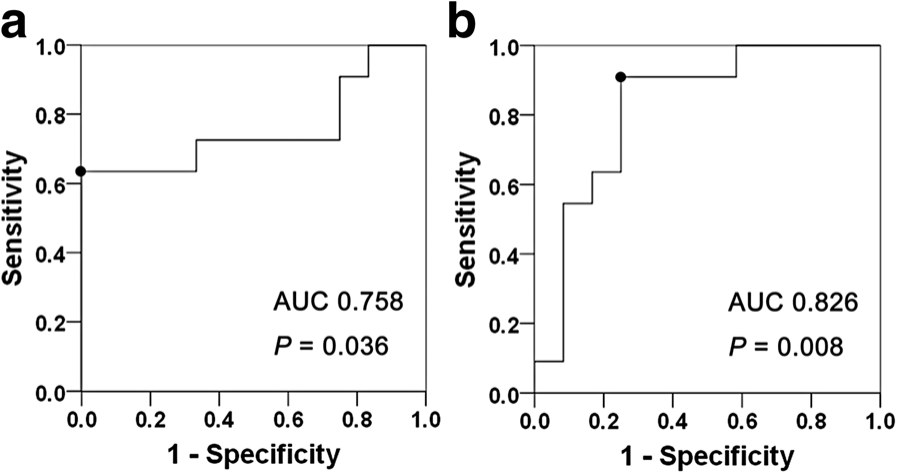
Receiver operating characteristic curve (ROC) analysis. **a** ROC curves to identify the appropriate estimated glomerular filtration rate (eGFR) cutoffs for the prediction of renal atrophy development show that eGFR <71.0 mL/min/1.73 m^2^ yields sensitivity of 63.6% and specificity of 100% (*circle*). **b** ROC curves to identify the appropriate serum IgE level cutoffs for the prediction of renal atrophy development show that serum IgE >436.5 IU/mL yields sensitivity of 90.9% and specificity of 75.0% (*circle*). *AUC* area under the curve

To help determine the ability of eGFR and serum IgE to reliably predict the development of renal atrophy after glucocorticoid therapy, and to identify their appropriate cutoffs, we used the ROC curve (Fig. 3). The area under the ROC curve for eGFR was 0.758 ± 0.111 (95% CI 0.539–0.976, *P* = 0.036). We found that eGFR <71.0 mL/min/1.73 m^2^ was the most appropriate cutoff that yielded sensitivity of 63.6% and specificity of 100% in predicting the development of renal atrophy (Fig. 3a). The area under the ROC curve for serum IgE was 0.826 ± 0.091 (95% CI 0.647–1.000, *P* = 0.008). Serum IgE >436.5 IU/mL was the most appropriate cutoff that yielded sensitivity of 90.9% and specificity of 75.0% in predicting development of renal atrophy (Fig. 3b).

## Discussion

We clarified that radiological abnormalities such as focal or diffuse renal cortical atrophy developed despite glucocorticoid therapy during a 2-year clinical course in about 50% of patients with IgG4-RKD with long-term follow up. In addition, we investigated the factors that determine whether or not radiologic abnormalities leave sequelae, and found that neither the number of involved organs nor serum IgG4 levels, but rather renal function such as eGFR and serum IgE at the time of initiation of glucocorticoid therapy, were related to the development of renal cortical atrophy.

In IgG4-RD, good responsiveness to glucocorticoid is characteristic regardless of which organs are affected [1, 2, 4–8, 10, 15, 17]. Until now, the response to glucocorticoids has been evaluated by means of identifying radiological abnormalities such as tumefactive or hyperplastic lesions, the clinical symptoms caused by them, and functional parameters of the affected organs, including renal function [8, 10], salivary secretory function [18], and pancreatic exocrine and endocrine function [19, 20]. Numerous reports reveal that these indicators improve at least initially after the initiation of glucocorticoid therapy. In type 1 AIP, the clinical picture and responsiveness to treatment have been investigated from the earliest period. In this disease, glucocorticoid therapy was reported to promptly achieve reduction of local or diffuse pancreatic swelling, improvement of clinical symptoms, and reduction of serum IgG4 levels [21]. Also in IgG4-RKD, it was shown that radiological abnormalities such as multiple low-density lesions, renal function, and frequently observed hypocomplementemia improved rapidly at the initial stage of glucocorticoid therapy [7, 8]. Similarly, an initial good response to glucocorticoid is generally recognized in the entire spectrum of IgG4-RD.

However, in contrast to the initial good response to glucocorticoids, recent studies investigating the long-term clinical course of IgG4-RD have revealed that dysfunction of the affected organs can persist. Transformation into chronic pancreatitis associated with pancreatic stones, atrophy, and exocrine and endocrine dysfunction has been reported in the long-term clinical course of type 1 AIP [19, 20]. A study investigating the long-term clinical course in IgG4-RKD showed that patients with pre-treatment eGFR under 60 mL/min/1.73 m^2^ calculated on the basis of the Japanese revised equation for estimating GFR from serum creatinine [22] attained not complete but only partial recovery of renal function one month after the start of glucocorticoid therapy, with residual dysfunction persisting thereafter, and that radiologically, focal or diffuse renal atrophy persisted [8]. Interestingly, renal lesions with complete recovery without any residual atrophy and others with atrophy coexisted radiologically under the circumstances in which global renal function, such as eGFR, improved after glucocorticoid therapy in this study, highlighting the heterogeneity among not only the affected organs but also individual lesions even within the same organ.

The heterogeneity of individual renal lesions in patients with IgG4-RKD was also identified histologically. Raissian et al. analyzed the clinicopathological features of 35 patients with IgG4-related tubulointerstitial nephritis (TIN), and found that the proportion of fibrosis to inflammation varied from specimen to specimen, with some specimens having variability in the extent of fibrosis and inflammation within each tissue sample [6]. In a study comparing light-microscopic findings of IgG4-RKD with those of other TIN in detail, Yoshita et al. also reported that all patients with IgG4-RKD had various fibrotic stages within each specimen, confirming the results of the Raissian study [23]. In our earlier study examining the renal histopathology of post-glucocorticoid treatment specimens, we found that in some lesions inflammatory cell infiltrates had disappeared without residual fibrosis, whereas other lesions developed persistent severe fibrosis [7]. This histopathological heterogeneity in the progression of fibrosis may explain why among lesions in which no radiological differences are detected, some recover completely without residual atrophy, whereas others become atrophic.

This study examined the factors related to the development of post-treatment renal cortical atrophy associated with fibrosis, and showed the possibility that pre-treatment renal function (eGFR) and serum IgE level could be factors predicting it. Both of these parameters differed significantly between the patients who developed renal atrophy after glucocorticoid therapy and those who did not. Age-adjusted, sex-adjusted, and serum IgG4 level-adjusted logistic regression analysis suggested that they were significantly related to development of renal atrophy. In addition, the usefulness of eGFR and serum IgE to predict the development of renal atrophy was suggested by our ROC curve analysis, in which the area under the ROC curve was 0.758 and 0.826, showing moderate and high accuracy, respectively. As the patients with eGFR <71.0 mL/min/1.73 m^2^ developed renal atrophy at specificity of 100%, an early initiation of glucocorticoid therapy seems reasonable before eGFR, which is calculated on the basis of CKD-EPI equations, declines to about 70 mL/min/1.73 m^2^. Considering the heterogeneity of each lesion in the progression of fibrosis, we can speculate that patients with lower pre-treatment eGFR had already developed more lesions with advanced fibrosis and, hence, such lesions resulted in irreversible atrophic changes despite glucocorticoid therapy. In drug-induced interstitial nephritis, a delay in treatment has been demonstrated to result in the development of fibrosis and worse renal outcomes [24]. As the International consensus guidance statement on the management and treatment of IgG4-RD recommends urgent treatment of IgG4-related tubulointerstitial nephritis to prevent irreversible renal failure [25], further study will be necessary to clarify the benefit of early initiation of glucocorticoid therapy in IgG4-RKD.

In addition to pre-treatment eGFR, pre-treatment serum IgE was also identified as a factor significantly related to development of renal atrophy in this study. In contrast to pre-treatment renal function, the impact of serum IgE elevation on renal prognosis has not thus far been documented in IgG4-RKD. Concerning the pathophysiology of IgG4-RD, however, upregulation of T helper (Th) 2 cytokines including interleukin (IL)-4 and IL-13, which promote IgE production, in the affected lesions have been reported [26–29]. Furthermore, it has been indicated that such Th2 cytokines, especially IL-13, play a dominant role in the fibrosis observed in Th2-upregulated conditions [30, 31]. On the other hand, Wallace et al. reported that baseline serum IgE elevations, in addition to elevations in serum IgG4 and blood eosinophil counts, could be useful predictors of relapses in patients with IgG4-RD treated with rituximab [32], suggesting the significant association between serum IgE and the clinical course or prognosis in this disease. In IgG4-RKD, although the precise etiology of elevated serum IgE remain unclear, it may reflect the upregulation of profibrotic Th2 cytokines that predispose patients to an increased risk of developing renal atrophy.

This study had several limitations. First, the treatment regimen and follow-up protocols were inconsistent among patients because of its retrospective and multi-institutional nature, complicating the evaluation of the influence of treatment protocol differences on patient outcome. Second, although this study included more patients with long-term, appropriate radiologic follow-up over 2 years than past ones, the number of patients was not sufficient to conclude the identified factors to be definitively significant predictors nor the indicated cutoffs to be optimal in this study. Therefore, larger-scale prospective studies will be needed to confirm our results.

## Conclusions

This study suggests that pre-treatment renal insufficiency and serum IgE elevation can be predictors of renal atrophy after glucocorticoid therapy, and that starting glucocorticoid therapy before eGFR declines to about 70 mL/min/1.73 m^2^ may be desirable in IgG4-RKD. Whether earlier initiation of therapy before renal function declines would more consistently prevent the development of renal atrophy remains to be confirmed through a larger-scale prospective study; nevertheless, these observations should be informative for establishing the optimal treatment strategy for IgG4-RKD.

## Additional file

Additional file 1: Table S1. Detailed data of 23 patients with IgG4-related kidney disease (PDF 213 kb)

## Abbreviations

3D: three-dimensional
ACEI: angiotensin converting enzyme inhibitor
AIP: autoimmune pancreatitis
ARB: angiotensin II receptor blocker
CI: confidence interval
CKD-EPI: Chronic Kidney Disease Epidemiology Collaboration
CRP: C-reactive protein
CT: computed tomography
eGFR: estimated glomerular filtration rate
IgE: immunoglobulin E
IgG: immunoglobulin G
IgG4: immunoglobulin G4
IgG4-DS: immunoglobulin G4 (IgG4)-related dacryoadenitis and sialoadenitis
IgG4-RD: immunoglobulin G4 (IgG4)-related disease
IgG4-RKD: immunoglobulin G4 (IgG4)-related kidney disease
IL: interleukin
OR: odds ratio
ROC: receiver operating characteristic
Th: T helper
TIN: tubulointerstitial nephritis
