# Supplementary material for: Factors related to renal cortical atrophy development after glucocorticoid therapy in IgG4-related kidney disease: a retrospective multicenter study
Source: Arthritis Res Ther. 2016 Nov 25;18:273. doi: 10.1186/s13075-016-1175-y (PMC5123425; doi:10.1186/s13075-016-1175-y)
Supplement: Additional file 1: Table S1. — Detailed data of 23 patients with IgG4-related kidney disease (PDF 213 kb) [file 13075_2016_1175_MOESM1_ESM.pdf]

## Table S1. Detailed Data of 23 Patients with IgG4-Related Kidney Disease

| No. | Follow-up Period (month) | Allergy | IgG (mg/dL) | IgG4 (mg/dL) | IgE (IU/mL) | Hypocomplementemia | CRP (mg/dL) | Cr (mg/dL) | eGFR (mL/min/ 1.73m$^2$) | Number of Extra-Renal Organs | Initial Dose of PSL (mg/kg/day) |
|---|---|---|---|---|---|---|---|---|---|---|---|
| 1 | 80 | + | 2680 | 1200 | 496 | + | <0.10 | 0.80 | 116.6 | 3 | 0.77 |
| 2 | 68 | + | 2250 | 785 | 411 | - | <0.10 | 0.40 | 114.8 | 2 | 0.97 |
| 3 | 29 | - | 1950 | 711 | 737 | - | 0.00 | 0.64 | 112.2 | 4 | 0.52 |
| 4 | 66 | + | 2630 | 751 | 870 | - | <0.10 | 0.60 | 104.8 | 1 | 0.71 |
| 5 | 59 | + | 4010 | 2160 | 680 | + | <0.10 | 0.70 | 101.9 | 4 | 0.49 |
| 6 | 29 | + | 1767 | 726 | 490 | - | <0.10 | 0.70 | 100.3 | 3 | 0.88 |
| 7 | 67 | + | 1810 | 671 | 113 | - | <0.10 | 0.90 | 98.5 | 2 | 0.57 |
| 8 | 93 | + | 2970 | 1330 | 419 | - | <0.10 | 0.80 | 95.1 | 3 | 0.53 |
| 9 | 34 | + | 2936 | 1070 | 17 | + | 0.00 | 0.72 | 92.5 | 4 | 0.61 |
| 10 | 31 | - | 3093 | 1290 | 8 | + | 0.10 | 0.74 | 92.2 | 4 | 0.59 |
| 11 | 91 | + | 5680 | 1920 | 248 | + | 1.17 | 0.90 | 90.6 | 2 | 0.65 |
| 12 | 42 | + | 2060 | 622 | 459 | - | <0.10 | 0.77 | 88.8 | 2 | 0.45 |
| 13 | 80 | - | 2256 | 984 | 292 | - | 0.10 | 0.59 | 88.4 | 4 | 0.34 |
| 14 | 61 | + | 3070 | 948 | 462 | - | <0.10 | 1.10 | 79.5 | 4 | 0.49 |
| 15 | 33 | - | 2938 | 1520 | 48 | - | 0.10 | 1.00 | 73.8 | 4 | 0.51 |
| 16 | 33 | + | 4171 | 2120 | 19 | - | 0.32 | 1.00 | 73.3 | 7 | 0.70 |
| 17 | 88 | - | 2850 | 1470 | 456 | - | 0.10 | 1.15 | 69.3 | 3 | 0.54 |
| 18 | 30 | + | 1923 | 263 | 502 | - | 0.30 | 1.00 | 65.6 | 2 | 0.52 |
| 19 | 28 | - | 2439 | 782 | 703 | - | 0.20 | 1.14 | 65.3 | 3 | 0.28 |
| 20 | 55 | + | 1756 | 408 | 513 | - | 0.20 | 1.20 | 64 | 3 | 0.31 |
| 21 | 30 | - | 6729 | 1630 | 454 | + | 0.90 | 1.74 | 30.9 | 3 | 0.73 |
| 22 | 64 | + | 3830 | 736 | 242 | + | 0.20 | 1.90 | 28.6 | 1 | 0.41 |
| 23 | 72 | - | 3695 | 486 | 1226 | + | 1.20 | 2.55 | 14.9 | 2 | 0.63 |

Note: Conversion factor for Cr: mg/dL to μmol/L, ×88.4.

Abbreviations: Cr, serum creatinine at diagnosis; CRP, serum C-reactive protein at diagnosis; eGFR, estimated glomerular filtration rate at diagnosis; IgG, serum immunoglobulin G at diagnosis; IgG4, serum immunoglobulin G4 at diagnosis; IgE, serum immunoglobulin E at diagnosis; PSL, prednisolone.
